# Multidimensional Feature Classification of the Health Information Needs of Patients With Hypertension in an Online Health Community Through Analysis of 1000 Patient Question Records: Observational Study

**DOI:** 10.2196/17349

**Published:** 2020-05-29

**Authors:** Aijing Luo, Zirui Xin, Yifeng Yuan, Tingxiao Wen, Wenzhao Xie, Zhuqing Zhong, Xiaoqing Peng, Wei Ouyang, Chao Hu, Fei Liu, Yang Chen, Haiyan He

**Affiliations:** 1 The Third Xiangya Hospital of Central South University Changsha China; 2 Key Laboratory of Medical Information Research Central South University College of Hunan Province Changsha China; 3 School of Life Sciences Central South University Changsha China; 4 School of Computer Science and Engineering Central South University Changsha China; 5 Information and Network Center Central South University Changsha China

**Keywords:** online health community, health information needs, patients with hypertension, physician-patient communication

## Abstract

**Background:**

With the rapid development of online health communities, increasing numbers of patients and families are seeking health information on the internet.

**Objective:**

This study aimed to discuss how to fully reveal the health information needs expressed by patients with hypertension in their questions in a web-based environment and how to use the internet to help patients with hypertension receive personalized health education.

**Methods:**

This study randomly selected 1000 text records from the question data of patients with hypertension from 2008 to 2018 collected from Good Doctor Online and constructed a classification system through literature research and content analysis. This paper identified the background characteristics and questioning intention of each patient with hypertension based on the patient’s question and used co-occurrence network analysis and the k-means clustering method to explore the features of the health information needs of patients with hypertension.

**Results:**

The classification system for the health information needs of patients with hypertension included the following nine dimensions: drugs (355 names), symptoms and signs (395 names), tests and examinations (545 names), demographic data (526 kinds), diseases (80 names), risk factors (37 names), emotions (43 kinds), lifestyles (6 kinds), and questions (49 kinds). There were several characteristics of the explored web-based health information needs of patients with hypertension. First, more than 49% of patients described features, such as drugs, symptoms and signs, tests and examinations, demographic data, and diseases. Second, patients with hypertension were most concerned about treatment (778/1000, 77.80%), followed by diagnosis (323/1000, 32.30%). Third, 65.80% (658/1000) of patients asked physicians several questions at the same time. Moreover, 28.30% (283/1000) of patients were very concerned about how to adjust the medication, and they asked other treatment-related questions at the same time, including drug side effects, whether to take the drugs, how to treat the disease, etc. Furthermore, 17.60% (176/1000) of patients consulted physicians about the causes of clinical findings, including the relationship between the clinical findings and a disease, the treatment of a disease, and medications and examinations. Fourth, by k-means clustering, the questioning intentions of patients with hypertension were classified into the following seven categories: “how to adjust medication,” “what to do,” “how to treat,” “phenomenon explanation,” “test and examination,” “disease diagnosis,” and “disease prognosis.”

**Conclusions:**

In a web-based environment, the health information needs expressed by Chinese patients with hypertension to physicians are common and distinct, that is, patients with different background features ask relatively common questions to physicians. The classification system constructed in this study can provide guidance to health information service providers for the construction of web-based health resources, as well as guidance for patient education, which could help solve the problem of information asymmetry in communication between physicians and patients.

## Introduction

### Background

Hypertension is currently the most common chronic disease and is a major risk factor for the morbidity and mortality of stroke and coronary heart disease in China [1]. According to statistics, the hypertension prevalence in Chinese residents aged 18 years or over is 27.9%, the hypertension awareness rate is 51.6%, and the hypertension control rate is 16.8% [2]. However, the hypertension control rate in the United States is close to 50% [3]. We urgently need to adopt measures to improve public awareness and health management of hypertension. An online health community is a health and medical online community of the *internet plus medical care* model, providing users with better knowledge services in the form of a community [4]. According to a research report, by the end of December 2018, the number of internet users in China had reached 820 million [5], and more than 190 million users had access to medical health information on the internet [6]. An online health community allows users to avoid long waits when seeking health information services, especially those with hypertension who need long-term self-health management at home. The health literacy of patients with hypertension in China is generally low. Owing to the small number of family physicians in the community and the low enthusiasm for signing contracts, the family physician policy has not been effectively promoted throughout the country [7]. After discharge, patients with hypertension cannot be guided or supervised by physicians to help manage the disease properly. When there are issues that may affect compliance, some patients will seek information or help on the internet. Wicks et al [8] showed that an online health community can help users benefit from managing their own disease symptoms, treating side effects, finding fellow patients, and asking for medication advice. We hope that information resource providers will effectively organize and recommend relevant information according to the information needs of each patient with hypertension. In the long term, this will help patients with hypertension reduce information asymmetry with physicians by personalized health education and improve their compliance. The research perspective of this study was to reflect the features of the information needs of patients with hypertension or their families in an internet-based environment according to the textual information disclosed by patients in an online health community when consulting with physicians.

### Related Studies

At present, many studies have been carried out on the health information needs of internet users in China and other countries. The investigators mainly used questionnaires [9-11], in-depth interviews [12], and content analysis methods [13,14] to study the types of health information needs of specific populations. The study populations included elderly individuals [9], college students [15], pregnant and parturient women [16], and other populations, and the health problems involved were diabetes [17], hypertension [18], cancer [19], depression [20], and others. Related studies have shown that different populations have different web-based health information needs, which change with the course of the disease [10,12,21]. Patients with different cancers have high needs for information on the likelihood of cure, survival, treatment side effects, and risk [19].

In recent years, with the rapid development of natural language processing research and machine learning algorithms, methods based on topic recognition and text mining have been gradually applied to the analysis of web-based health information needs. Compared with the questionnaire survey method, the web text mining method can fully consider a patient’s expression and idioms from a user’s perspective. The obtained demand characteristics are more in line with the user’s real information needs [22]. Chen used the k-means method to conduct clustering analysis on the text of three online health communities and found that different communities had different hot topics, as well as the same topics, such as patient experience, treatment, drugs, and body management [23]. Lv used the expectation maximization clustering method to perform topic analysis, member role analysis, and sentiment analysis on the MedHelp website and finally defined seven hot topics, including detailed personal introduction, emotional support, symptoms, examinations, complications, medication, and treatment [24]. Patrick et al [25] used a public dataset to predict the intent of the questioner and extracted patients’ opinions, emotions, countermeasures, and social support topics from the data of patients with cancer and mental illness. Zhang [26] suggested that the background information of users, such as health status and age at the time of asking questions, should be considered in research on patient information needs. Owing to the professionalism and complexity of medical language and the issues of web users’ descriptions, such as miswriting and colloquial words, it is difficult to manage user-generated content, and the machine learning algorithm model that it relies on needs to be trained and validated in an annotated corpus [27,28]. However, a Chinese corpus for patient consultation after current annotation is still lacking.

There are already some studies on the classification of health questions. However, these studies focused on information needs for health care professionals [26,28,29]. Guo et al [30,31] constructed a Chinese health question classification corpus to study topics on the information needs of patients with hypertension, but these studies did not fully consider the background information of patients with hypertension, such as disease history, symptoms and signs, medication, gender, and age. As a result, they failed to reflect the features of the health information needs of more sophisticated patients with hypertension.

In summary, with the text information actually published in an online community as the research object, the obtained data can better reflect the real situation of the health information needs expressed by patients with hypertension during online consultations. However, nonmanual annotation methods, such as latent Dirichlet allocation (LDA) topic recognition, still have some deficiencies in Chinese medical text applications and cannot effectively reflect the semantic similarities of medical concepts. Although the LDA algorithm can identify the required topics from a large amount of text information, owing to the complexity and professionalism of the medical language, the feature keywords of the topic need to be manually summarized after generating the topic model. The final generated topic tags are medicine dependent, and expert discussions are designed to summarize the expressions of the most typical words [32]. Therefore, in this study, we used manual coding to analyze web text content.

### Study Aim

In this study, on the basis of previous studies, we constructed a multidimensional classification system of the health information needs of patients with hypertension according to their consultation records in an online health community and used multidimensional features to reveal the features of the health information needs of patients with hypertension in a web-based environment.

## Methods

### Data Collection and Preprocessing

Data collection in this study was based on a search engine in the Chinese medical field [33] to filter nonreal, noneffective, and nonauthoritative medical information. Considering that the search engine can aggregate patient consultation data from different websites with disease keywords, it was convenient for us to obtain web-based consultation data for patients with hypertension. In addition, the search engine can mark the consulted physician, the physician’s title, and the level of the medical institution. In this study, we searched for relevant records with *hypertension* as the keyword in the search engine and obtained 10,000 related records from 2008 to 2018. Most of the records provided by the search engine were from Good Doctor Online [34]. Good Doctor Online is one of China’s leading web-based medical platforms. As of December 2019, it has information on 610,000 physicians from 9917 regular hospitals in China [34]. A survey of Good Doctor Online found that when consulting a physician, patients submit content according to a certain information description framework as follows: consultation title, disease, description of the condition, previous treatment status and effect, and how to get help. Considering that the resources under the same website have similar structures, in this study, we only included 8338 nonduplicate consultation records available on the website. A total of 1000 consultation records were randomly selected for further analysis, and the C# program (developed by YFY within the Key Laboratory of Medical Information Research, Central South University, College of Hunan Province, PR China) was used to extract information about the records of patient questions available on the website.

### Multidimensional Classification System Construction

We initially summarized the knowledge framework of hypertension from the Guidelines for the Prevention and Treatment of Hypertension in China [35], the 8th Edition of Internal Medicine, and the Health Education Reader Book for Hypertensive Patients. Among them, the Guidelines for the Prevention and Treatment of Hypertension in China is the latest guidance document in China. With reference to the guideline development process of the World Health Organization and the Chinese Medical Association, the compilation team established a set of guidelines for the prevention and intervention, diagnosis and evaluation, classification and stratification, and treatment management of hypertension with Chinese characteristics according to the practice of hypertension prevention and treatment in China.

Based on the knowledge of hypertension, we initially constructed a system involving 12 categories, including epidemiology, etiology and pathogenesis, pathophysiology and pathology, clinical manifestations and complications, laboratory tests, diagnosis and differential diagnosis, prognosis, treatment, hypertension in special populations, risk factors, hazards, and management.

**Figure 1:**
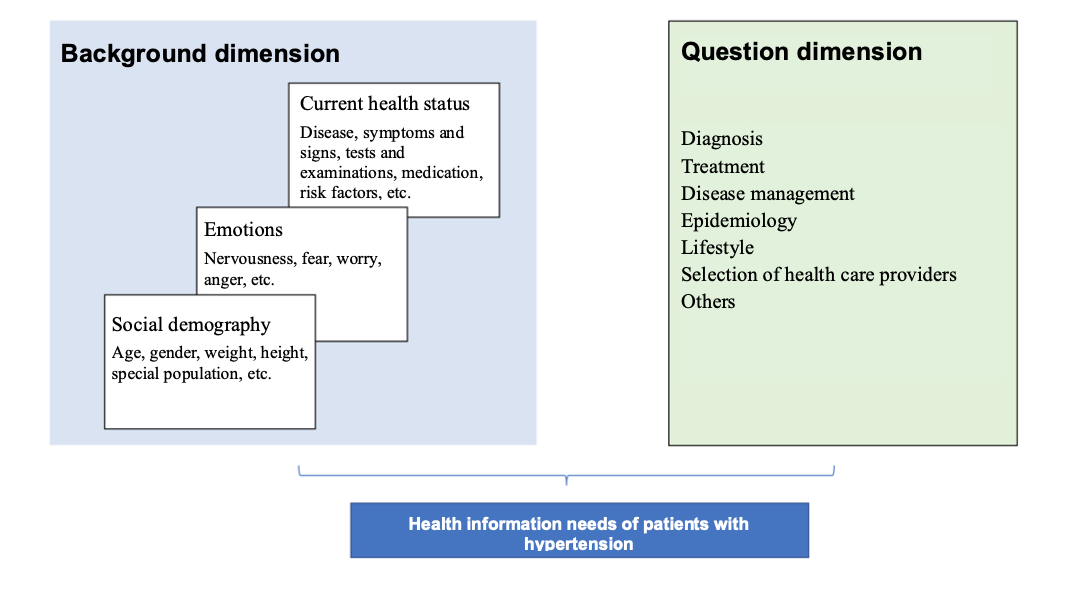
Classification framework diagram for the health information needs of patients with hypertension.

Thereafter, we took into consideration the above-mentioned general clinical question classification system [29], hierarchical model of public health information query scenario, and knowledge elements of hypertension prevention and treatment guidelines; read and summarized the consultation records (Figure 1); and initially constructed a multidimensional classification framework diagram for the health information needs of patients with hypertension. The classification framework mainly included the background dimension system (social demography, emotions, and current health status) and the question dimension system (diagnosis, treatment, disease management, epidemiology, lifestyle, selection of health care providers, and others).

### Annotation Process

Initially, two annotators with medical informatics backgrounds independently classified and annotated 100 records randomly selected from the 1000 consultation records mentioned according to the preliminary classification system and the classification example of each category. In the annotation process, each annotator proposed the experience of the annotation process of various entities, made the records, and finally completed the classification table and annotation descriptions for the health information needs of patients with hypertension after reaching a consensus through consultation. Thereafter, the two annotators randomly selected 200 records from the remaining records for repeat annotation, and they discussed inconsistent records (n=23) and reached a consensus after discussion. Finally, the two annotators completed the annotation of the remaining 700 questions and answers independently (ie, 350 questions and answers each). We manually aligned the entities of each type after the annotation was completed. Specifically, the original words were aligned with standard terms. An investigator with a background in medical informatics used this classification system for annotation. The classification system also included a series of annotation rules to improve the usability and accuracy of the classification system.

**Figure 2:**
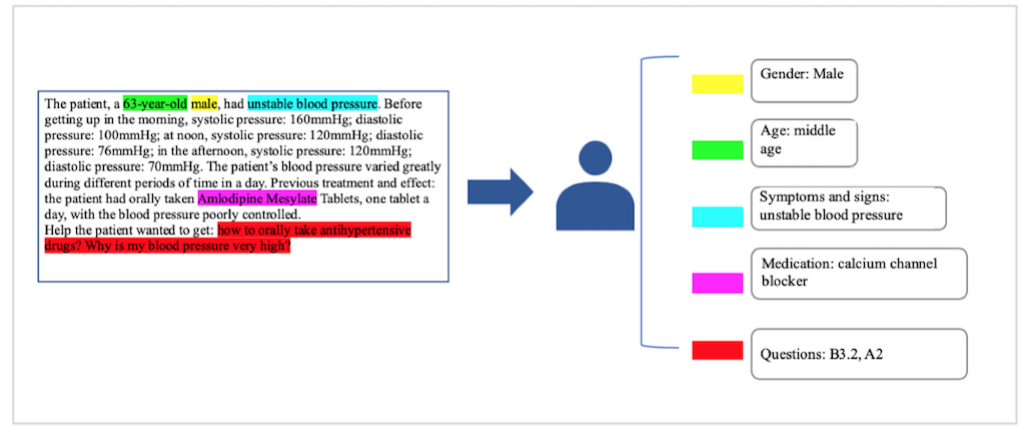
Structured content coding for the original question record of a patient with hypertension. B3.2 denotes a question about taking medication and A2 denotes a question about symptoms and signs.

In particular, we coded each record and aligned the patient description terms with medical phrases under the joint guidance of two medical information professionals and one medical professional. We completed a structured representation of the original question records for patients with hypertension in the sample set. An example is shown in Figure 2.

### Co-Occurrence Analysis and Clustering Analysis

Co-occurrence analysis is one of the content analysis methods in bibliometrics. This method assumes that there is some inherent relationship between multiple keywords that appear in the same document. Co-occurrence frequency refers to the number of occurrences of a group of words in the same document. A larger co-occurrence frequency of two keywords indicates a closer relationship between the two words. Cluster analysis of these words based on co-occurrence frequency can reflect the association between multiple words [36]. Co-occurrence of keywords was considered to be the main approach for identifying research themes and knowledge structure by displaying the relationship between keywords [37]. This study used the co-occurrence analysis and clustering analysis methods to study the relationship between multiple questions from patients with hypertension on the internet. The representative k-means algorithm was selected in the cluster analysis.

This study used the integrated development environments PyCharm and RStudio under Windows, and Python3.6 and R language (developed by ZRX within the Key Laboratory of Medical Information Research, Central South University, College of Hunan Province, PR China) as programming languages for data analysis. In the co-occurrence analysis, the NumPy package was used to calculate the co-occurrence matrix of patient question codes, and the Circlize package was used to visually present the co-occurrence network between question codes. Besides, we used the Sklearn package to implement the k-means clustering algorithm in the cluster analysis.

## Results

### Feature Mining of the Background Dimension System

The background features of patients with hypertension can be divided into the following eight dimension types: disease, tests and examinations, symptoms and signs, drugs, risk factors, emotions, lifestyles, and demographic data. Among them, risk factors are summarized from the medical knowledge of hypertension, which intersects with other dimensions. The main contents include obesity, high salt and low potassium diet, genetics, diabetes, kidney disease, mental and psychological factors, smoking, drinking, pregnancy, hyperlipidemia, snoring, and others. The feature distribution of background dimensions in 1000 records is shown in Table 1. The drug dimension appeared most frequently in the sample set (814/1000, 81.40%), followed by symptoms and signs (795/1000, 79.50%), tests and examinations (563/1000, 56.30%), demographic data (527/1000, 52.70%), diseases (499/1000, 49.90%), risk factors (352/1000, 35.20%), emotions (119/1000, 11.90%), and lifestyles (114/1000, 11.40%).

Feature mining of the background dimension system can reflect the personalized features of the health information needs of every patient with hypertension on the internet. The personalized features cover 355 names of drugs, 395 names of symptoms and signs, 545 names of tests and examinations, 526 kinds of demographic data, 80 names of diseases, 37 names of risk factors, 43 kinds of emotions, 6 kinds of lifestyles, and 71 kinds of questions. In addition, for the demographic data, we built a system of rules. As shown in Table 2, based on the rules, we effectively mined a total of 385 patient gender records from the text (male: 178, female: 206), with gender unknown in other records. Moreover, we effectively mined a total of 352 age records, including seven underage patients, 177 young patients, 116 middle-aged patients, and 48 elderly patients. It can be seen that basic patient information disclosed on the internet is not complete, although it is important to mine patients’ health information needs. To our knowledge, users currently ask physicians to provide sociodemographic information, such as age and gender, when consulting for disease-related issues on Good Doctor Online. However, this part is only visible to physicians and patients. Sociodemographic factors can be obtained by manually labeling the content according to the labeling rules from the public consultation data submitted by patients. However, the multidimensional classification system introduced in this study can make up for the incomplete or missing data of a single dimension through multidimensional mining.

**Table 1:** Frequency distribution of the background dimension system.

Dimension name	Keywords	Quantity	Records (N=1000), n (%)
Drugs	Calcium channel blocker, angiotensin II receptor antagonist, β blocker, angiotensin-converting enzyme inhibitor, traditional Chinese medicine, antihypertensive drugs, amlodipine besylate, Western medicine, compound antihypertensive drugs, diuretics, and aspirin enteric-coated tablets	355	814 (81.40%)
Symptoms and signs	Dizziness, headache, unstable blood pressure, chest discomfort, discomfort, giddiness, numbness, heart discomfort, pain, rapid heartbeat, movement disorder, palpitation, fatigue, weakness, and head swelling	395	795 (79.50%)
Tests and examinations	Physical examination, electrocardiogram examination, laboratory test, computed tomography examination, color Doppler ultrasound examination, cardiac color Doppler ultrasound examination, B-ultrasound examination, blood lipids, blood glucose, renal function, and routine blood test	545	563 (56.30%)
Demographic data	Gender, age, kinship, medical history, weight, height, fertility, occupation, family history, and others	526	527 (52.70%)
Diseases	Cerebrovascular disease (cerebral infarction, cerebral thrombosis, or cerebral hemorrhage), cardiovascular disease (coronary heart disease or heart disease), digestive system disease (fatty liver), rheumatic disease, other vascular disease, respiratory disease, endocrine disease, urinary tract systemic disease, and pregnancy-induced hypertension	80	499 (49.90%)
Risk factors	Dyslipidemia, diabetes, heredity, kidney disease, nervousness, fertility, drinking, obesity, stress, high blood glucose, smoking, emotional problems, anxiety, and menopause	37	352 (35.20%)
Emotions	Fear, doubt, worry, nervousness, vexation, irritability, anxiety, discomfort, exhaustion, moodiness, stress, bewilderment, depression, and emotional instability	43	119 (11.90%)
Lifestyles	Diet, exercise, sleep, weight loss, drinking, smoking, and others	6	114 (11.40%)

**Table 2:** Distribution of identified demographic data of the patients with hypertension.

Attribute		Quantity	Records (N=1000), n (%)
**Gender**			**385 (38.50%**)
	Female	206	
	Male	178	
**Age**			**352 (35.20%)**
	Underage (<18 years)	7	
	Young (18-44 years)	177	
	Middle-aged (45-64 years)	116	
	Elderly (over 65 years)	48	

### Feature Mining of the Question Dimension System

The constructed question classification system for patients with hypertension included 49 items in seven categories, including diagnosis, treatment, disease management, epidemiology, lifestyle, selection of health care providers, and others. Owing to space limitations, only the distributions of the categories in the first and second classes of the classification dimension system are presented (Table 3).

Among 1000 web-based consultation records, 738 (73.80%) had patient questions that were related to treatment (B), mainly drug treatment (B3; quantity: 472), including drug selection, other drug-related questions, drug side effects, medication time, mode, and others. Among them, other drug-related questions were mainly about user consultation on whether medication is needed under the current situation. Regarding questions on medication time, there was a focus on long-term medication or drug discontinuation. Although it is widely known that patients with hypertension must take antihypertensive drugs for the rest of their life, they still asked this question repeatedly with anxiety and worry. Consultation about the side effects of drugs mainly involved drug safety, drug contraindications, and side effects.

Additionally, 32.30% (323/1000) of patients asked questions about the diagnosis of a disease (A), including seeking interpretation of clinical findings and recommendation of test items. Some patients also asked physicians questions about general diagnosis (A0) (eg, whether they contracted a disease). Moreover, 41.00% (410/1000) of patients consulted about not only medication (B1). Furthermore, 20.40% (204/1000) of patients asked questions about epidemiology (D1), such as the etiology of the disease and correlation between the disease and signs and symptoms, and their needs for information were not limited to the diagnosis and treatment of the disease. Most medical workers paid little attention to answering these questions, and they mainly focused on the process of medical services. In addition, 4.80% (48/1000) of patients consulted about what they should pay attention to in daily life (E0) in order to maintain a good lifestyle, including diet (E4), weight loss (E1), exercise (E2), emotional management (E3), and many other aspects. Some patients realized that hypertension is a chronic disease and that a bad lifestyle could contribute to the progression of hypertension. They wanted to improve their health through a good lifestyle. Further, 10.00% (100/1000) of patients consulted about the selection of health care providers (F0), and they mainly focused on whether they should go to the hospital (F3) or choose hospitalization (F4) according to their physical conditions and other questions. Some patients asked questions about the impact of the disease on fertility (G1) and the reliability of their opinions. A total of 27 patients asked physicians about the reliability of opinions they saw on the internet or were told by others. Seven questions were classified into G0 (others-others) as they could not be classified into other categories.

**Table 3:** Distribution of second-class codes of question features.

First category, second category		Quantity	Records (N=1000), n (%)
**A: Diagnosis**			**323 (32.30%)**
	A0: Other diagnosis-related questions	97	
	A1: Tests and examinations	113	
	A2: Symptoms and signs	176	
	A3: Diagnostic cost	2	
**B: Treatment**			**778 (77.80%)**
	B1: Not only drug treatment	410	
	B2: Nondrug treatment	29	
	B3: Drug treatment	472	
C: Disease management		128	135 (13.50%)
**D: Epidemiology**			**204 (20.40%)**
	D1: Etiology of a disease	27	
	D2: Severity of a disease	41	
	D3: Age at which a disease occurs	3	
	D4: Others	74	
	D5: Prognosis of a disease	75	
**E: Lifestyle**			**48 (4.80%)**
	E0: Other lifestyles	18	
	E1: Weight loss	4	
	E2: Exercise	11	
	E3: Emotion management	3	
	E4: Diet	21	
**F: Selection of health care providers**			**103 (10.30%)**
	F0: Other selection of health care provider-related questions	19	
	F1: Department selection	7	
	F2: Outpatient service	7	
	F3: Physician selection	37	
	F4: Hospital selection	40	
**G: Others**			**66 (6.60%)**
	G0: Others	7	
	G1: Fertility	31	
	G2: Medical insurance	2	
	G3: Opinion judgment	27	

### Feature Mining of Co-Occurrence in the Question Dimension System

**Figure 3:**
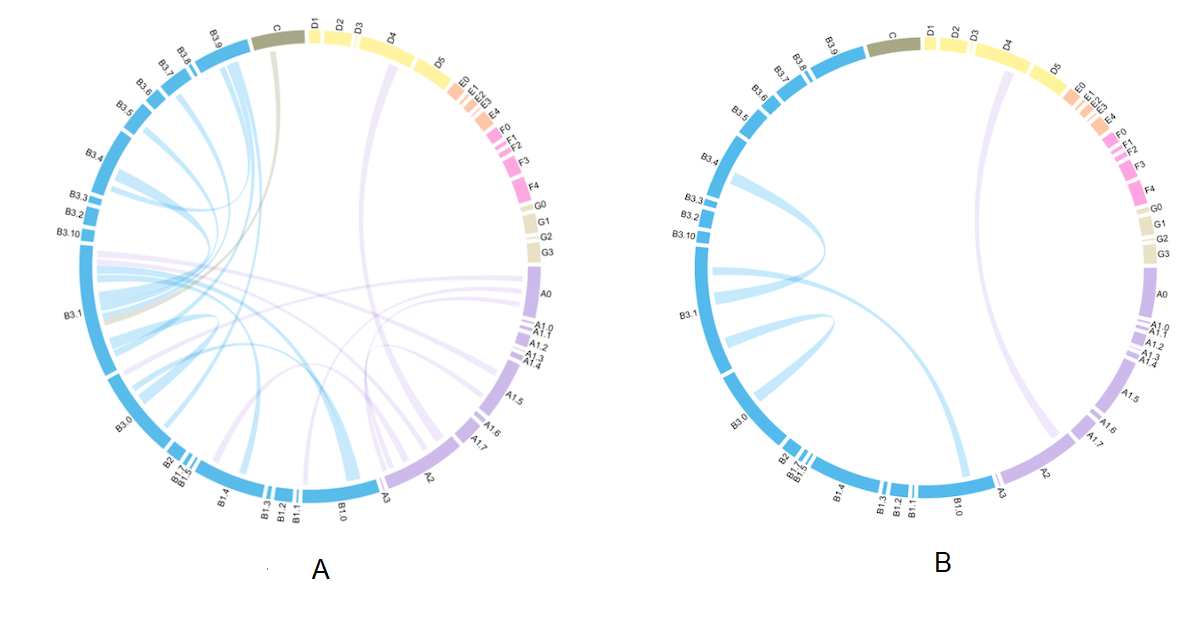
Co-occurrence graph for question type. (A) Co-occurrence frequency ≥20%; (B) Co-occurrence frequency ≥30%.

Since each patient’s online consultation contains multiple questions and involves various information needs, co-occurrence network analysis based on the question type was conducted according to the question features. A total of 658 patients with hypertension asked physicians several questions from one online consulting record. Treatment (B) and diagnosis (A) were the most common issues among patients (Figure 3). Because patients with hypertension need to take drugs for a long time, 283 patients who consulted physicians on the internet were very concerned about drug selection (B3.1). In addition, they asked other treatment-related questions (B), including side effects (B3.4), whether to take the drug (B3.0), how to treat the disease (B1.0), etc. Moreover, among 176 patients who consulted physicians on the internet about the causes of clinical findings (A2), 39 consulted physicians about the relationship between clinical findings and the disease (D4).

### Feature Mining for Patients With Hypertension According to Background and Question Dimensions

We performed k-means clustering on all classification features of the consultation. After many tests, we selected question type as the feature variable, and the elbow method determined that the clustering model was the best when k was 7. Finally, we obtained a schematic diagram of seven types of questions for patients with hypertension. The medical concepts expressed by the main features of each type were clearly defined (Table 4). The seven categories of the population contained background details, such as test and examination items, dizziness symptoms, and calcium channel blocker use.

The first category of the population was “how to adjust medication,” which included patients who mainly needed information about drug selection. The background details of their questions mainly involved information on the names of drugs, including calcium channel blockers, angiotensin Ⅱ receptor antagonists, angiotensin converting enzyme inhibitors, and β-receptor blockers. The second category was “what to do,” which included patients who mainly asked physicians to provide information on disease management and prevention. There were more female patients in this group. The third category was “how to treat,” which included patients who were mainly seeking information from physicians on disease treatment. Their background descriptions focused on the use of calcium channel blockers and dizziness symptoms. The fourth category was “phenomenon explanation,” which included patients who consulted physicians about the causes of clinical findings, the correlation between clinical findings and a certain disease, and the treatment plan of the disease. Their background descriptions mainly included tests and examinations, and dizziness and headache. Most of them were female patients who used calcium channel blockers and angiotensin Ⅱ receptor antagonists. The fifth category was “test and examination,” which included patients who mainly consulted physicians about tests and examinations. Their background data mainly included the description of tests and examinations, and dizziness. Most of them were young male patients who used calcium channel blockers. The sixth category was “disease diagnosis,” which included patients who mainly consulted physicians about whether they had a certain disease, whether they needed drugs, the causes of symptoms and signs, and their treatment. Their background data mainly included information of tests and examinations, and dizziness. Most of them were young patients. The seventh category was “disease prognosis,” which included patients who mainly asked physicians questions such as “Can the disease be cured?” and “How to treat it?” Their background data mainly included tests and examinations, dizziness, brain disease, and calcium channel blocker use.

**Table 4:** Classification of the health information needs of patients with hypertension.

Category	Main question, category statistics (N=1000)	Examples of typical questions	Description of main background features (N=1000)	Quantity
1	B3.1 (n=185); B3.0 (71); B3.4 (61)	How to adjust medication or change drugs? Do I need medication? Does the medicine have side effects?	Calcium channel blockers (n=124), angiotensin Ⅱ receptor antagonists (74), angiotensin-converting enzyme inhibitors (61), dizziness (59), tests and examinations (56), and β-receptor blockers (54)	262
2	C (69)	What to do and how to prevent it?	Tests and examinations (64), female (53), dizziness (47), and calcium channel blockers (45)	205
3	B1.0 (110); B1.4 (68)	How to treat it? How to treat a disease?	Calcium channel blockers (48) and dizziness (46)	184
4	A2 (97); D4 (24); B1.4 (20)	Why do I have such a symptom? What is the relationship between a disease and a symptom? How to treat a disease?	Tests and examinations (36), dizziness (31), female (28), calcium channel blockers (28), headache (24), and angiotensin Ⅱ receptor antagonists (23)	97
5	A1.5 (53); A1.7 (31)	What examinations are needed? What does the result of the examination mean?	Tests and examinations (66), male (26), dizziness (26), youth (19), and calcium channel blockers (19)	94
6	A0 (85); B3.0 (19); A2 (18); B1.0 (18)	Have I got a disease? Do I need medication? Why do I have such a symptom? How to treat it?	Tests and examinations (32), dizziness (21), and youth (19)	85
7	D5 (73); B1.0 (16); B1.4 (15)	Can this disease be cured? How to treat it? How to treat this disease?	Tests and examinations (29), dizziness (19), brain disease (19), and calcium channel blockers (17)	73

## Discussion

### Principal Findings

We found some common and distinct features of information needs from the consultation records of patients with hypertension in an online health community. In this study, we constructed a multidimensional *background plus question* classification system for mining various features of health information needs and effectively revealed what Chinese patients with hypertension want to communicate with physicians online. According to our knowledge, no researchers have studied the classification of health information needs in background and question dimensions among patients with hypertension. Our constructed classification system underlines the personal features of the information needs of patients with hypertension, which could help online information service providers and relevant researchers personalize their services. Related online health information resources can be identified according to the classification system of hypertension information needs in this study so as to make online health information resource organization more refined and meet the needs of users.

We found that drug information is quite important to Chinese patients with hypertension on the internet. Additionally, 81.40% (814/1000) of them described their previous medication situation when consulting physicians on the internet, and there are many types of drugs involved. Moreover, 73.80% (738/1000) of them asked their physicians how to treat the disease. This is similar to the findings of Guo et al [30,31,38]. Chinese patients with hypertension mainly asked physicians about drug treatment, including drug selection, drug side effects, medication time, and mode. The results of Zhong et al [39] also showed that the medication literacy of discharged patients with hypertension was poor. More than 70% of patients had no substantial knowledge of the effects and side effects of the medications they were taking. It is worth noting that 16.10% (161/1000) of the questioners with hypertension were concerned about deeper information, including the prognosis, etiology, and progression of the disease.

Most background information described by patients with hypertension when they consulted a physician on the internet involved drug information, followed by symptoms and signs, which accounted for about 80% of patient records. More than 49% of patients mentioned details, such as tests and examinations, diseases, and sociodemographic data. To our knowledge, these values have only been revealed in our study. In this study, we found that patients with hypertension were most concerned about treatment (738/1000, 73.80%) and diagnosis (313/1000, 31.30%). This is similar to the findings of Guo et al [38]. It is worth noting that 16.10% (161/1000) of patients with hypertension were concerned about deeper information, such as the prognosis, etiology, and progression of the disease. In a web-based consultation, Chinese patients with hypertension usually ask physicians many questions without significant priorities, but in an offline consultation, physicians often fail to answer some questions patients are concerned about owing to the lack of time and the long line of waiting patients.

In this study, we also found common associations when patients asked questions. This is different from the findings of Guo et al [30], and it has not been reported in previous studies. Clustering results based on question features can be explained by medical knowledge. Patients with hypertension can be classified into seven categories, including “how to adjust medication,” “what to do,” “how to treat,” “phenomenon explanation,” “test and examination,” “disease diagnosis,” and “disease prognosis.” When new patients with hypertension ask questions, health information service providers can predict which type of population the patients belong to using the clustering model and can recommend other relevant information that the patients may need while recommending the question information. However, owing to the complexity and uniqueness of questioners’ background information, we tried to combine the background and question features to conduct clustering research and association rule mining research; however, we did not obtain an interpretable clustering model. This also reflects the personalized differences in the real health information needs of patients with hypertension.

Some patients with hypertension in China still lack an accurate understanding of the risk factors of hypertension and need to acquire more effective knowledge. The risk factors for hypertension were involved in 35.00% (350/1000) of consultations in this study. The risk factors appearing in the research results were presented by the patients when describing the problem, and they may not necessarily recognize the factors as risk factors. Those risk factors were also not question intentions at that time. We hope that when patients consult physicians on the internet, future internet tools will be able to highlight the risk factors of patients with hypertension in a timely manner, as well as promptly remind and push health intervention knowledge of corresponding diseases and their risk factors to patients with hypertension. This is conducive to the provision of targeted information services by information resource providers and to meet the personalized goals of patient education. For example, it can promptly and continuously alert obese patients with hypertension to take measures to control their weight.

The patients with hypertension included underage, young, middle-aged, and elderly people, and thus, hypertension in China can occur in people of all ages. In future health information services, the information that is more in line with the actual health situation and that can improve the health status could be screened from dimensions, such as population and risk factors. In addition, some women were worried about the effects of drugs on pregnancy when they asked about medication. At present, the second-child policy has been widely implemented in China. Medical service personnel need to pay special attention to pregnant women, such as women with hypertension, help them give birth to healthy babies under the support of the existing medical resources, strengthen their accurate understanding of hypertension from the perspective of health information services, and help them relieve their anxiety.

In our study, we found that manual consultation analysis of patient consultation text in Chinese is still needed. Among patient consultation records, medical terms, such as drugs, symptoms and signs, tests and examinations, and diseases, are often not standardized in the patient information description. Owing to typographical errors, omissions, ambiguous words, etc, manual alignment or correction is still needed. This was also noted in the article by Guo et al [30]. The glossary of terms accumulated in this study on summarizing the wording habits of patients in consultations will improve the accuracy of machine learning in feature recognition so as to obtain more accurate predictions of needs categories.

Through this study, we hope to strengthen patient participation with the help of internet technology for improving the personalized characteristics of existing patient education. Patient engagement in their care plays an important role in improving health outcomes [40]. With patient participation, patient education can provide detailed information about drugs, symptoms and signs, tests and examinations, demographic data, diseases, risk factors, emotions, lifestyles, and other information identified in the consultation records for each patient in real time. The cluster analysis method extracts the corresponding population characteristics of patients and further provides the information that the patients need or may need. The process of providing health information to patients through a platform has actually begun to provide health education to the patients, that is, when patients are puzzled about health problems and submit questions on the internet, they can respond to health education services with the help of network technology. Personal questioning data have accumulated on web-based platforms, which have formed the growing consulting big data of users in online health communities. With the help of these big data and the cluster analysis method, it is possible to identify the subdivided hypertension group and obtain the generally needed health knowledge to help the construction and continuous update of patient health education content. We hope that in the future, internet tools will become individual physician assistants for patients under the supervision of professionals. For the information needs of patients, health education can be effectively carried out in real time.

In this study, we used a multidimension approach to identify the features of the health information needs of patients with hypertension in a web-based environment when consulting a physician. Thereafter, according to the classification of these needs, we effectively organized and constructed web-based health information resources and accurately matched the web-based health information resources with the health information needs of each questioner through identification. The unmet information needs will be the focus of future web-based health information resources. In future studies, we will determine how to assess the degree of matching between health information needs and web-based health information resources. In the future, we will also determine how to apply this classification system to clinical consultations in order to reduce information asymmetry between physicians and patients and to help patients and physicians manage diseases together.

### Limitations

The classification system may have limitations owing to the insufficient sample size for analysis. In the future, we should continue to expand the sample size and carry out a larger classification of health information needs. The sample data were from a single website, and all information was from the web-based consultation records available on the website. Thus, the target group of the website may affect the results of the construction of the classification system. In the future, we will collect data from several health websites and conduct comparative studies. The information needs of patients with hypertension in this study were the information needs expressed by patients. However, this study did not fully address the feature recognition of patients’ unexpressed information needs. Our next goal is to determine how to improve compliance in patients with hypertension.

### Conclusions

The classification system of hypertension information needs involving the *background plus question* approach constructed in this study reflects the personalized features of the health information needs expressed by patients with hypertension to physicians in a web-based environment. We believe that multidimensional health information needs should have more refined identifiers, which will enable patients with hypertension to fully express their health information needs during a web-based consultation, thus allowing relevant information service providers of online health communities to organize information resources related to hypertension according to these identifiers. They can use the classification system of hypertension information needs constructed in this study as a classification basis to organize web-based health information resources and provide more accurate information push services to patients when they consult physicians over the internet or wait for physicians to reply in order to reduce information asymmetry in communication between physicians and patients. On the other hand, the Chinese corpus of patients with hypertension based on artificial coding in this study will lay the foundation for future research on the features of automatic identification of health information.

## Abbreviations

LDA: latent Dirichlet allocation
